# Research on steel surface defect classification method based on deep learning

**DOI:** 10.1038/s41598-024-58643-1

**Published:** 2024-04-08

**Authors:** Yang Gao, Gang Lv, Dong Xiao, Xize Han, Tao Sun, Zhenni Li

**Affiliations:** 1 The State Key Laboratory of Rolling and Automation, Northeastern University, Shenyang, 110819 China; 2 Information Science and Engineering School, Northeastern University, Shenyang, 110819 China

**Keywords:** Steel surface defect detection, YOLOv5, Attention mechanism, BiFPN, Engineering, Mechanical engineering

## Abstract

Surface defects on steel, arising from factors like steel composition and manufacturing techniques, pose significant challenges to industrial production. Efficient and precise detection of these defects is crucial for enhancing production efficiency and product quality. In accordance with these requisites, this paper elects to undertake the detection task predicated on the you only look once (YOLO) algorithm. In this study, we propose a novel approach for surface flaw identification based on the YOLOv5 algorithm, called YOLOv5-KBS. This method integrates attention mechanism and weighted Bidirectional Feature Pyramid Network (BiFPN) into YOLOv5 architecture. Our method addresses issues of background interference and defect size variability in images. Experimental results show that the YOLOv5-KBS model achieves a notable 4.2% increase in mean Average Precision (mAP) and reaches a detection speed of 70 Frames Per Second (FPS), outperforming the baseline model. These findings underscore the effectiveness and potential applications of our proposed method in industrial settings.

## Introduction

In numerous nations, the steel industry stands as a pivotal pillar, underpinning the economic vitality of the country. Within this industry, strip steel products find extensive applications in various sectors including construction engineering, everyday hardware, and automobile manufacturing, thereby exerting a substantial impact on the national economy. In the year 2020, China's annual production of crude steel surged to an impressive 1.053 billion tons, constituting a commanding 57% share of the global output of crude steel. Despite a marginal dip in China's crude steel production in 2021, it still maintains a majority stake, surpassing the 50% mark, and continues to represent half of the global production, firmly establishing itself as the largest consumer of iron ore worldwide^1^. Presently, steel reigns supreme as the most prevalent structural and functional material globally, boasting the lowest emissions and the highest recycling rate throughout its life cycle among all metal materials. Its invaluable contributions to the advancement of human civilization are immeasurable^2^.

To uphold product excellence and mitigate losses and expenses stemming from quality issues, enterprises undertake real-time assessments of steel surfaces, ensuring early identification and rectification of surface imperfections. Traditional approaches for steel surface defect detection predominantly encompass manual visual spot inspections, magnetic flux leakage detection, and infrared detection. These methods offer the advantages of simplicity and cost-effectiveness; however, their limitations are stark, necessitating substantial manpower and time investment. They heavily rely on the expertise and discernment of personnel, carrying a risk of human error in judgment. Additionally, given the typically intricate environment of steel production facilities, replete with disturbances such as noise and dust, workers must conduct inspections under suboptimal conditions. This can lead to fatigue and erroneous assessments, ultimately falling short of meeting the requisites of high-volume, high-speed, and high-efficiency production. Addressing these demands mandates the adoption of more intelligent and efficient technological means for defect detection.

In the initial phases of research, scholars predominantly relied on feature extraction-based methods like edge detection and image segmentation to discern steel surface defects. In 2009, Yang^3^ conducted a comprehensive study on the multi-classification and multi-feature complex pattern recognition challenge in strip surface defect detection. He outlined an integration of classifiers and hierarchical classifier technology, proposing a combination of boosting algorithm and Supervised Learning of Incomplete data using Query-based learning(SLIQ) decision tree for strip surface defect identification. Through this classifier recognition approach, the recognition accuracy exceeded 92%, markedly enhancing classification precision. In 2011, Sadeghi^4^ et al. devised a technique that swiftly and accurately identifies such defects by employing two-dimensional wavelets for normalized and non-normalized assessments of local image values, confined within the limit of [0,1]. Through the normalization of resulting partial image variances and the incorporation of variance variation values with relevant partial images, this approach outperforms other methods in terms of correct detection of defective areas. Nevertheless, these techniques necessitate manual feature design, demanding distinct feature extraction methodologies for varying defect types, consequently constraining the algorithm's portability and adaptability.

The number of research using deep learning technologies to identify surface flaws in steel has increased recently. Deep learning algorithms possess robust capabilities for automatic learning and discrimination. They exhibit a remarkable proficiency in discerning defects of diverse shapes, sizes, colors, and other attributes, delivering high-accuracy identification that remains impervious to environmental and human factors. This facilitates automated, high-speed detection processes, significantly reducing inspection duration and enhancing production efficiency. Additionally, deep learning algorithms have the capacity for continuous refinement of their recognition prowess through ongoing learning and iteration. They can adeptly adapt to varying production environments and different defect types, ensuring a high level of reliability.

In 2012, Jonathan^5^ introduced a steel defect classification method founded on a maximum pooling convolutional neural network. Using this technique, supervised feature extraction is carried out straight from the pixel description of steel flaw images. The results demonstrate that even in the absence of prior knowledge, outstanding outcomes can be achieved, surpassing classifiers designed for fault identification in textured materials that were trained using widely used feature descriptors. In 2014, Soukup et al.^6^ proposed a method for detecting steel surface flaws using photometric stereoscopic pictures and convolutional neural networks(CNN). A CNN was trained on a set of photometric stereoscopic images, specifically track flaws, that displayed metal surface flaws. This method elucidates the correlation between defects and non-defects by comparing the anticipated reflective properties of surface defects. These defects manifest as cavities on the rail surface and serve as indicators of impending surface deterioration, possibly culminating in rail fracture.

Object detection stands as a pivotal domain within computer vision, dedicated to identifying objects within images or videos while ascertaining their precise locations and respective categories. Initial object detection algorithms relied predominantly on handcrafted features and classical methods for machine learning, including boosting and support vector machines (SVM), among others. In 2014, Chu et al.^7^ introduced a method by using improved dual support vector machines to categorize strip surface flaws. This approach leveraged density information to guide the pruning of expansive training datasets, added samples without labels to training datasets that were sparse and refined the Twin Support Vector Machine (TWSVM). Ultimately, the results of studies performed on the strip surfaces flaw database show the effectiveness of TWSVM. These methodologies necessitated extensive manual feature engineering and parameter fine-tuning, often constrained by the computational power and storage capacity of computers. The difficulties were not effectively tackled until the development of deep learning algorithms.

Target detection has changed dramatically since deep learning algorithms were developed, shifting from traditional classification and regression problems to a comprehensive strategy utilizing deep neural networks. Target detection has become far more accurate and robust as a result of this paradigm change. Notably, it allows for the direct learning of features from the original image, obviating the need for manual feature design. In 2016, Amu et al.^8^ applied the Region-CNN(R-CNN) algorithm to identify defects in railway freight car wheels. This algorithm employs selective search technology for detection, segmenting the image (X-Ray) into regions, and subsequently subjecting these regions to CNN feature processing. This approach aids in identifying various types of wheel defects, along with pinpointing the specific defect locations. In 2018, A real-time technique for detecting strip surface flaws was presented by Li et al.^9^ using an enhanced YOLO detection network. They refined the network to make the YOLO completely convolutional. This enhanced network furnishes a comprehensive solution for strip surface flaws inspection. In 2020, Wei et al.^10^ proposed an enhanced Faster R-CNN network for steel surface flaw detection. To mitigate the risk of overlooking a multitude of small defects, they employed a weighted Region of Interest (RoI) pool instead of a conventional RoI pool, yielding significant performance enhancements. Additionally, they incorporated deformed convolution to capture more sporadic faults and reduce the number of false-positive boxes by strictly implementing Non-Maximum Suppression (NMS). In 2021, Tang et al.^11^ proposed a flaw recognition technique leveraging multi-scale maximum pooling (MSMP) and the attention principle. As the baseline, they built a two-stage detection network using a pre-trained ResNet50 network and introduced the attention principle along with the MSMP module. At every ResNet50 stage, the attention principle enhances the retrieved feature maps, enabling the model to concentrate on regions pertinent to final detection results, while disregarding ineffective or even detrimental background areas. The MSMP method gradually enlarges the receptive field, discerning the most crucial contextual features, thereby substantially enhancing detection accuracy. Peng et al.^12^ harnessed deep learning techniques in conjunction with the YOLOv4 model to propose an improved version for detecting surface flaws in automotive steel. They employed K-means clustering to generate anchor frames and introduced the Squeeze-and-Excitation(SE) attention module into the network to augment the feature extraction capacity for small targets. The MSFT-YOLO network was presented in 2022 by Guo et al.^13^ and was designed for industrial scenarios characterized by significant image background interference and vast variations in flaw size. Through the incorporation of the Transformer-based TRANS component into the network's detecting head and backbone, they achieved the amalgamation of features and global information. Moreover, they employed a multi-scale feature fusion structure to amalgamate features of varying scales, fortifying the detector's adaptability for targets of different sizes. Chen et al.^14^ proposed a fast detection network for surface defects in strip steel based on deformable convolution and attention mechanism (DCAM Net). By integrating deformable convolution, the receptive domain of the defect feature extraction network was extended, and then a coordinated attention (CA) module was introduced to further decompose the pooling operation, effectively improving the detection efficiency of surface defects. Luo et al.^15^ proposed a camouflaged defect detection network (CDDNet). They designed a lightweight recurrent decoupled fully connected attention (RDFCA) with cost-effectiveness calculation and a new adaptive scale-equalizing pyramid convolution(ASEPC), which utilizes inter-layer feature correlation to achieve cross-scale feature fusion. Li et al.^16^ proposed a multipath feature aggregation network based on YOLOv5 that can significantly improve the performance of steel defect detection. Although the accuracy has been improved to a certain extent, the speed of defect detection is very low.

In light of the prevailing challenges in steel defect detection within the contemporary steel industry, this work explores deep learning-based techniques for detecting steel surface flaw. The following highlights the main contributions of this study:

1. Comparative analysis of YOLO variants: The study conducted a comprehensive comparison of the detection performance of three distinct iterations of YOLO models namely, YOLOv5, YOLOX, and YOLOv7, utilizing the NEU-DET dataset. Considering both overall detection accuracy and processing speed, it was found that YOLOv5 proves to be relatively more apt for this specific task.
2. Innovative YOLOv5-KBS network: To address challenges posed by extensive background interference in images and substantial variations in defect sizes, the paper introduces a novel architecture, the YOLOv5-KBS network, building upon the foundation of YOLOv5. This enhancement integrates the Squeeze-and-Excitation (SE) model into the core backbone and improves the Path Aggregation Network (PANet) into the BiFPN. Comparatively, the final YOLOv5-KBS model exhibits a noteworthy increase of 4.2% in mAP over the baseline model. Additionally, it achieves a detection speed of 70 FPS, signifying distinct advantages and offering valuable insights for future research and applications.

## Methods

### YOLOv5

The You Only Look Once (YOLO) network stands as a single-stage target detection approach, demonstrating superior performance at the same scale when compared to other algorithms^17–19^. Introduced by Glenn Jocher in 2020, YOLOv5^20^ boasts commendable detection speed and continues to undergo continuous enhancements. It is available in four versions: s, m, l, and x. For the purposes of this study, YOLOv5l was selected for experimentation.

**Figure 1:**
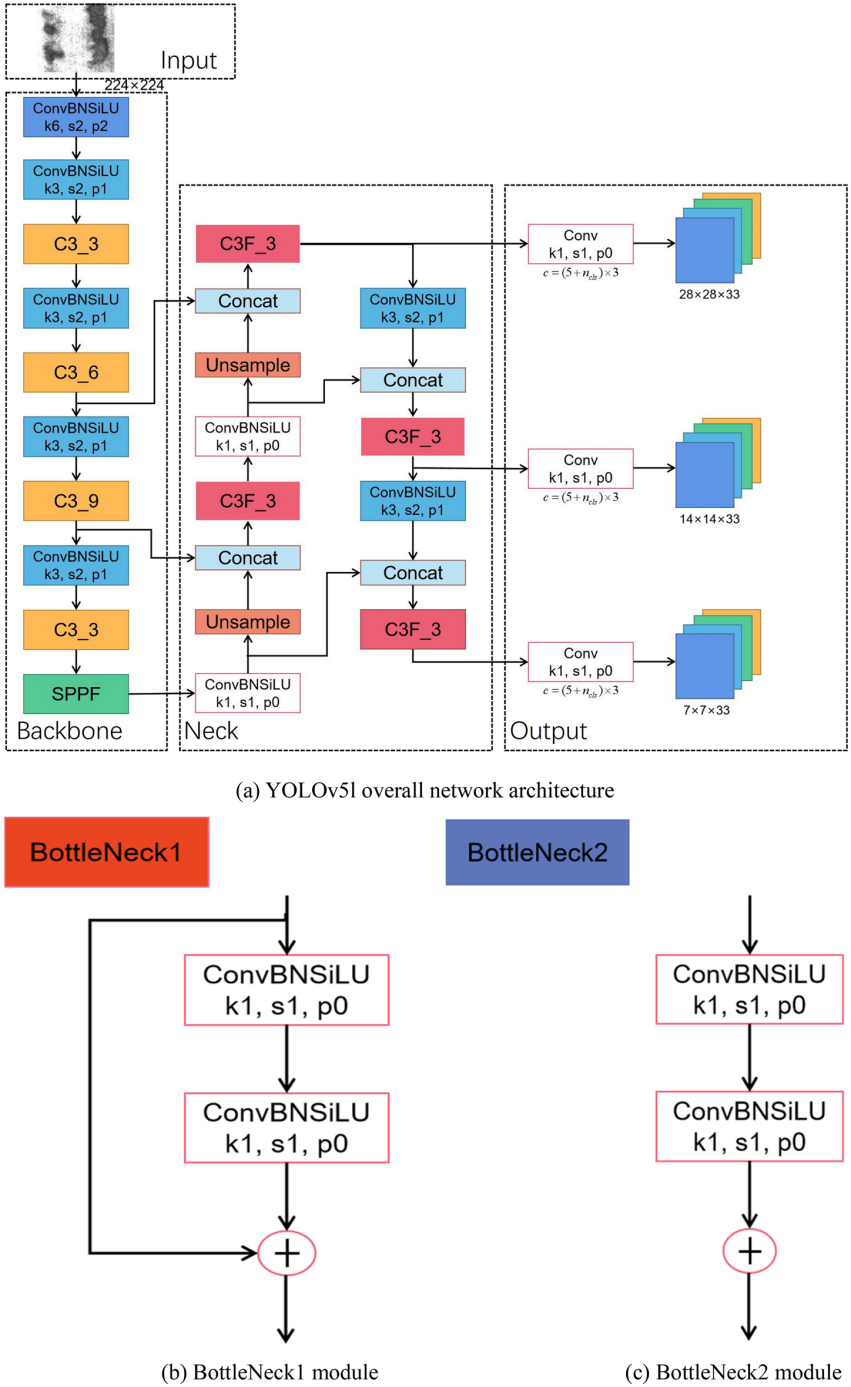

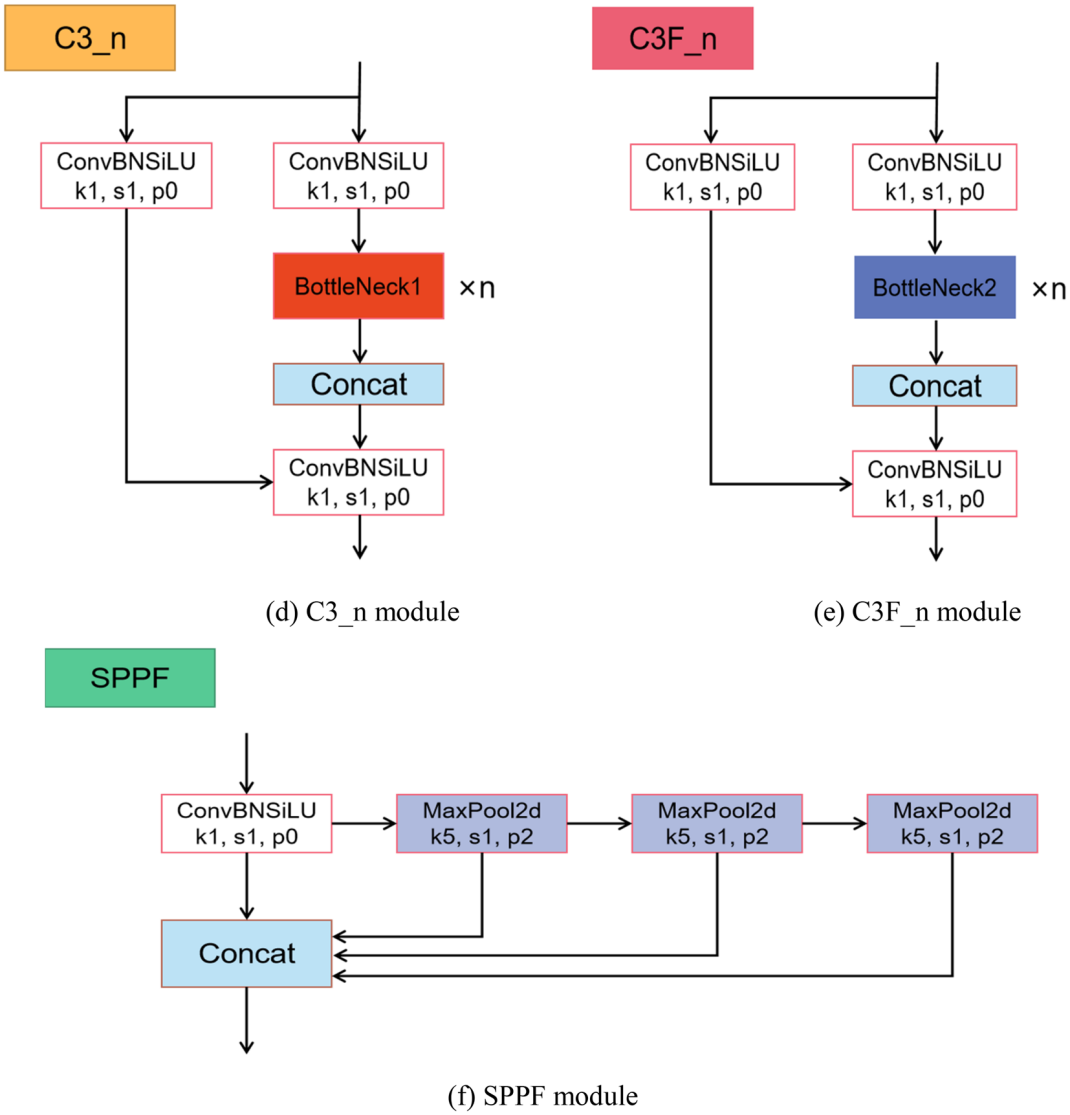
YOLOv5l and each module architecture (k, s and p respectively represent kernel size, stride and padding. Numbers represent the corresponding size).

The network architecture of YOLOv5 can be segmented into four key components: input, backbone, neck, and head. The model's structure and components for YOLOv5l are depicted in Fig. 1.

*Input* The input stage comprises two primary components: data preprocessing and data augmentation. Data preprocessing involves transforming input images into a tensor form that the model can process. YOLOv5 employs a technique called letterboxing for data preprocessing, which resizes the image to the required dimensions while maintaining its aspect ratio. This may lead to parts of the image being bordered with black, which can be disregarded or trimmed in subsequent steps. Regarding data augmentation, YOLOv5 adopts a range of techniques that encompass cropping, flipping, rotating, deforming, and more. They generate diverse image samples to augment the dataset and enhance the model's performance. Additionally, YOLOv5 incorporates a technology called MixUp, enabling the fusion of two images into one during training. This aids in enhancing the model's generalization ability.

*Backbone* The backbone network employed here is a variation of the deep residual network (ResNet). Specifically, YOLOv5 adopts the Cross Stage Partial Network (CSPNet) structure for its backbone, which is an adaptation that splits the ResNet's residual block into two segments. This division serves for memory consumption of the model. The input maps of features are divided into two sections by the CSPNet architecture: one section is utilized for the residual block computation, while the other acts as a link between the input and the output. This design allows the model to learn more meaningful feature representations. The Spatial Pyramid Pooling-Fast (SPPF) module is devised to address the challenges posed by large computational requirements and sluggish processing speeds in the original Spatial Pyramid Pooling(SPP) module. In particular, SPPF employs three 5 × 5 maximum pooling cores in succession to replace the original 5 × 5, 9 × 9, and 13 × 13 maximum pooling cores of the SPP module. This modification serves to further enhance processing speed while retaining the core functionalities of the original module.

**Figure 2:**
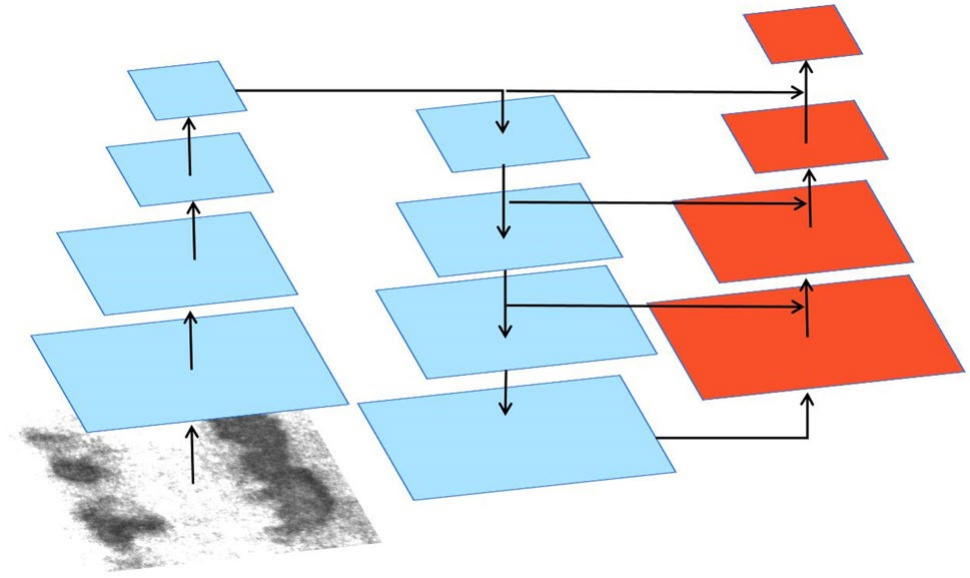
PANet architecture.

*Neck* The Neck section constitutes a feature fusion network primarily tasked with amalgamating multi-scale feature maps derived from the backbone network, thereby enhancing the accuracy and efficiency of target detection. A pivotal technique employed here is the PANet (Path Aggregation Network), which stands as a feature pyramid network tailored for target detection applications. The structural overview of PANet is shown in Fig. 2. PANet establishes connections between feature maps at various levels and merges them through a process called path aggregation. During this aggregation, PANet employs a top-down propagation of features to acquire more refined semantic information upwards, while simultaneously leveraging bottom-up detailed information for downward propagation. This methodology results in the acquisition of richer and more precise feature representations across diverse scales, consequently amplifying the performance of target detection.

*Head* The Head component constitutes a pivotal segment of the YOLOv5 target detection network. It leverages output feature maps with dimensions of 7 × 7, 14 × 14, and 28 × 28 for target detection. The section employs 1 × 1 convolutions to increase the total amount of channels within the map features of various scales obtained in the Neck component. Within the Head, the three detection layers respectively align with the three distinct-sized feature maps acquired in the Neck component. Each feature map is preconfigured with three anchors of varying aspect ratios. These anchors are instrumental in both predicting and refining the position information and classification of the targets.

### K-means clustering

Anchor boxes serve a crucial function in both the training and prediction phases. During the training phase, the network initially calculates the loss between the predefined anchor boxes and the actual boxes, and then iteratively adjusts to better fit the actual boxes. In the prediction phase, the model generates a fixed-size anchor box on the image requiring detection. Once it identifies the category, it refines the prediction box parameters to more accurately encompass the target. Hence, selecting the right anchor boxes helps the model run faster in addition to improving accuracy.

The default anchor boxes in YOLOv5 are derived from clustering on the COCO dataset. However, steel surface defects exhibit considerable differences in size and shape compared to the objects in the COCO dataset. As a result, these default anchors are unfit for our dataset. To address this, this study employs the K-means clustering method, utilizing Intersection Over Union(IOU) instead of Euclidean distance to measure the distance between the sample and the cluster. This approach is employed to effectively cluster the anchor boxes.

To be specific, the K-means clustering algorithm proceeds as follows:

1. Set the desired number of clusters (in this article's case, it's set to 9). Randomly select 9 samples from the entire dataset to serve as the initial cluster centers.
2. Once the cluster centers are established, calculate the distances between each sample and the clusters. This information is used to assign each sample to the nearest cluster.
3. After the sample classification is completed, recompute the cluster centers based on the samples assigned to each cluster.
4. Repeat steps two and three until the termination conditions are met or the cluster centers do not change. This signifies the convergence of the algorithm.

### Attention model

**Figure 3:**
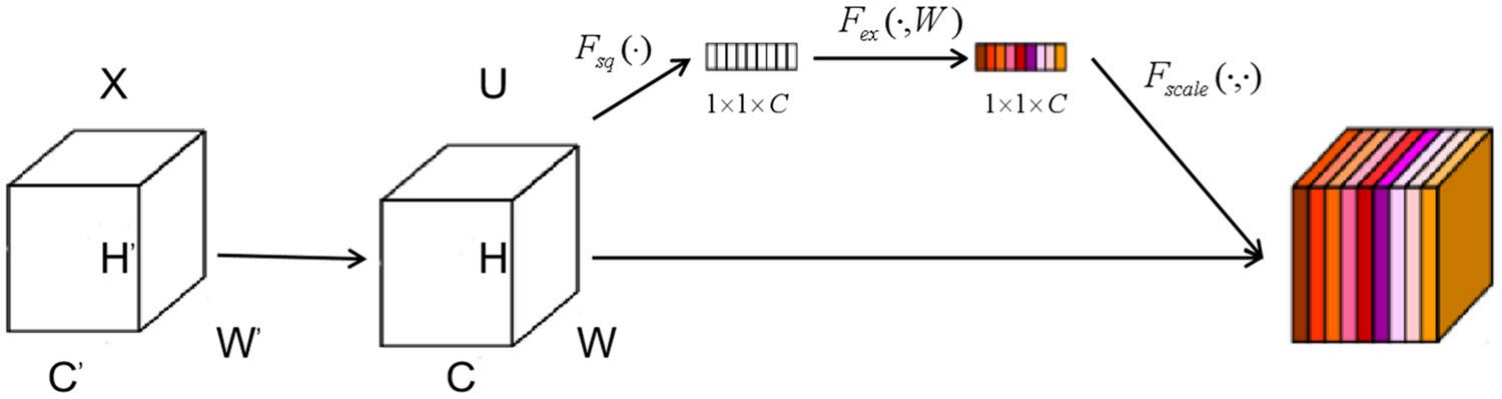
Internal structure of SE module (C. H and W represent the channels for input features, height and width).

The Squeeze-and-Excitation (SE) Network was introduced by Hu et al. in 2018^21^. Its main goal is to characterize the interactions between the network's convolutional feature channels explicitly to improve the standard of the presentations that the network produces. Through the use of this process, the network can acquire the capability to incorporate whole information, allowing it to amplify valuable features while downplaying less pertinent ones. The architecture of the SE attention module is depicted in Fig. 3.

As illustrated in Fig. 3, the input X is transformed into feature U, which is then recalibrated using the constructed SE module. Feature U initially undergoes a squeeze operation, wherein the feature map of dimensions H × W is compressed to a size of 1 × 1, serving as a channel descriptor. Following the squeeze (F_sq_) operation, an activation process is applied to generate weights for each channel through an activation function. Finally, by applying these weights to U, the result generated by the SE module is achieved. It's worth noting that the SE module assumes distinct roles at various depths within the network.

### Bidirectional feature pyramid network

**Figure 4:**
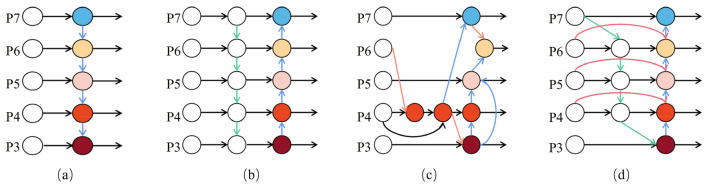
Structure of different feature pyramid networks (P3–P7 represents multi-scale features).

The Bidirectional Feature Pyramid Network (BiFPN) was introduced by Tan et al. in the paper "EfficientDet" in 2020^22^. The Feature Pyramid Network (FPN)^23^ was the earliest feature fusion architecture utilized to integrate feature maps of varying scales from top to bottom. With ongoing research, the advent of networks like PANet^24^ and NAS-FPN has paved the way for the development of more cross-scale feature fusion networks. However, these networks don't discriminate between different feature inputs during the fusion process; they simply perform straightforward fusion operations. Recognizing that different input feature maps contribute differently to the output feature map, BiFPN tackles this challenge by incorporating learnable weights into the network. These weights enable the network to discern the significance of different input features. The structures of different feature fusion networks are shown in Fig. 4.

The traditional FPN is constrained by a one-way information flow, as it solely propagates information in a single direction. To address this limitation, PANet introduced a bottom-up path to allow for bidirectional information flow. While NAS-FPN aims to fuse feature maps across scales to incorporate more features, this approach results in a more complex model structure, making it challenging to interpret and modify, and imposing higher hardware requirements. In contrast, BiFPN borrows the cross-scale fusion operation of NAS-FPN but simplifies the model architecture. It eliminates nodes with only one input feature and restricts cross-scale fusion from occurring within the same layer. This simplification allows for the fusion of as many features as possible while maintaining a more manageable and interpretable model structure.

In BiFPN, each input contains an additional weight. Currently, there are three popular weighted fusion techniques: boundless fusion, softmax-based fusion, and rapid standardized fusion. To optimize runtime performance, BiFPN chooses the rapid standardized fusion method. Its calculation process is shown in Eq. (1)^22^.

1 $$\begin{array}{c}O=\sum_{i} \frac{{w}_{i}}{\varepsilon +\sum_{j} {w}_{j}}\cdot {I}_{i}\end{array}$$

where $\varepsilon$ is a very small number used to avoid numerical instability, ${I}_{i}$ is the input feature, and ${w}_{i}$ is a learnable value.

Finally, BiFPN accomplishes bidirectional cross-scale feature fusion alongside fast normalized fusion. This is exemplified in the feature fusion at level 6, as depicted in Eqs. (2) and (3)^22^.

2 $$\begin{array}{c}{P}_{6}^{td}={Conv}\left(\frac{{w}_{1}\cdot {P}_{6}^{in}+{w}_{2}\cdot {Resize}\left({P}_{7}^{in}\right)}{{w}_{1}+{w}_{2}+\varepsilon }\right)\end{array}$$

3 $$\begin{array}{c}{P}_{6}^{out}={Conv}\left(\frac{{w}_{1}^{\prime}\cdot {P}_{6}^{in}+{w}_{2}^{\prime}\cdot {P}_{6}^{td}+{w}_{3}^{\prime}\cdot {Resize}\left({P}_{5}^{out}\right)}{{w}_{1}^{\prime}+{w}_{2}^{\prime}+{w}_{3}^{\prime}+\varepsilon }\right)\end{array}$$

Among them, ${P}_{i}^{td}$ is the intermediate feature of the level $i$ and ${P}_{i}^{out}$ is the output feature of the level $i$. $Conv$ represent convolution operations and add batch normalization and activation functions after each convolution.

### Proposed methods

**Figure 5:**
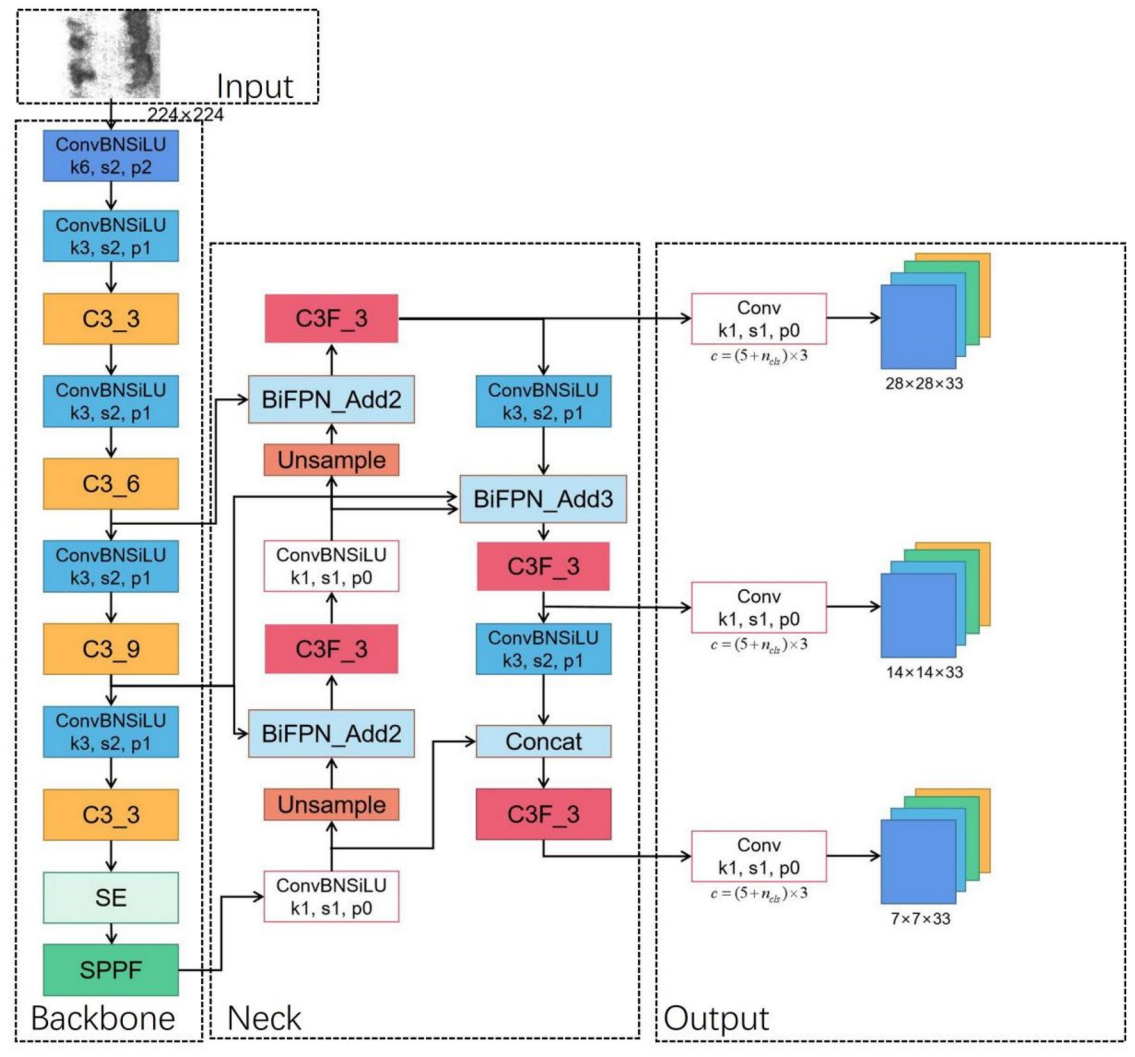
YOLOv5-KBS model.

The main enhancements we propose are made to the YOLOv5l base model. To make the model more capable of identifying larger flaws such as scratches, the SE attention module is integrated into the last layer of the Backbone. Due to the smaller feature map size, this strategic placement preserves a richer set of high-level semantic features relevant to larger targets. Furthermore, we replace PANet with the BiFPN to improve feature fusion capabilities. The final refined structure, represented as YOLOv5-KBS, is shown in Fig. 5.

## Experimental and discussion

### Data description and process

The NEU-DET dataset, which is accessible to the public, was used in this study to analyze steel surface flaws. This dataset was gathered by Northeastern University using a line array camera within a factory setting. Each image measures 200 × 200 pixels and encompasses six distinct defect types: cracks, inclusions, plaques, pitting, oxide skin penetration, and scratches. Corresponding labels in the dataset are designated as crazing, inclusion, patches, pitted_surface, rolled-in_scale, and scratches. The dataset comprises a sum of 1800 pictures across all categories, with 300 images allocated to each category. For the purpose of this article, the dataset is split in a 9:1 mix to training sets which are employed for model training, and test sets which are utilized for model evaluation.

The anchor boxes resulting from the re-clustering of the NEU-DET dataset using the method outlined in "K-means clustering" section are presented in Table 1.

**Table 1:** Anchor boxes obtained by re-clustering.

Feature map size	Anchor box size
7 × 7	[(20, 40), (26, 84), (50, 47)]
14 × 14	[(64, 83), (192, 31), (36, 209)]
28 × 28	[(143, 76), (81, 148), (177, 215)]

Indeed, the dataset in this work employs the XML format for annotation. As YOLOv5 operates with annotation information stored in text files (usually in TXT format), a conversion is necessary for compatibility. This conversion process allows the dataset to be effectively utilized with the YOLOv5 model. It is vital to ensure that the annotation information remains consistent and accurate throughout the conversion process. This guarantees that the converted TXT format annotation data can be appropriately employed with the YOLOv5 algorithm, optimizing training and inference.

In the realm of data augmentation, YOLOv5 adopts an array of techniques to bolster the model's resilience and generalization capabilities. These methods encompass operations such as cropping, flipping, rotating, and deforming, among others. By applying these operations, a diverse set of image samples is generated, augmenting the volume of data and enhancing the model's overall performance. Additionally, YOLOv5 leverages a technique called MixUp. During training, MixUp merges two images into one, heightening the model's generalization prowess. This technique is instrumental in enhancing the model's adaptability to a wider array of scenarios.

### Experimental condition

In the experimental setup, all algorithms were implemented based on the PyTorch framework. The utilized graphics card is the NVIDIA A16. To mitigate overfitting, data augmentation techniques like rotation and splicing were applied to the input images. The specific training parameters are detailed in Table 2.

**Table 2:** Training parameter settings.

Training parameters	Value
Learn rate	0.01
Momentum	0.937
weight_decay	0.0005
image_size	224 × 224
Epochs	100

### Evaluation metrics

This article predominantly employs the mean Average Precision (mAP) and Frames Per Second (FPS) to gauge the detection accuracy and speed, respectively. The precision and recall of the model are intricately linked with the mAP. Precision signifies the ratio of accurately identified instances of a specific type of anomaly. Recall, on the other hand, denotes the proportion of identified anomalies by the model against the actual total of defects correctly detected. The precision and recall can be succinctly expressed through Eqs. (4) and (5)^25^.

4 $$\begin{array}{c}{\text{Precision}} \, =\frac{TP}{\left(TP+FP\right)}\end{array}$$

5 $$\begin{array}{c}{\text{Recall}} \, =\frac{TP}{\left(TP+FN\right)}\end{array}$$

Among these categories, TP denotes the positively classified samples, FP refers to the negatively classified samples misidentified as positive, FN signifies the positively labeled samples misclassified as negative, and TN denotes the accurately classified negative samples.

### Model comparison experiment

We selected and compared different baseline models YOLOv5, YOLOX^26^ and YOLOv7^27^. Under the above environment, set the same hyperparameters for the NEU-DET dataset to train the models. Subsequently, the models were evaluated on the test set to conduct a comprehensive analysis of their detection capabilities. The detection findings are summarized in Table 3.

**Table 3:** Effects of different models.

	YOLOv5	YOLOX	YOLOv7
Crazing	**0.537**	0.320	0.465
Inclusion	**0.836**	0.732	0.811
Patches	0.895	0.885	**0.912**
pitted_surface	0.734	**0.821**	0.721
rolled-in_scale	0.668	0.495	**0.688**
Scratches	0.874	0.828	**0.924**
mAP	**0.757**	0.678	0.753
FPS	**71**	3	55

Significant values are in bold.

Examining Table 3, it is evident that in terms of detection accuracy, YOLOv5 demonstrates superior performance in detecting steel surface defects. It achieves an impressive average mAP of 75.7%, surpassing the other two models. Particularly in the identification of crazing and inclusion, YOLOv5 exhibits remarkable proficiency, showcasing its prowess in detecting smaller targets. Conversely, YOLOv7 exhibits a heightened proficiency in recognizing defects like scratches, thereby displaying a slightly enhanced ability to identify larger targets.

When it comes to detection speed, YOLOv5 stands out as the frontrunner. Its Frames Per Second (FPS) reaches an impressive 71, outpacing the other two models. However, due to the decoupling of the head in YOLOX, it experiences a reduction in detection speed, resulting in a lower FPS, which may not meet the demands for high-speed detection in industrial settings. Despite YOLOv7 boasting an FPS of 55, a capability suitable for industrial applications, when considering a holistic evaluation of detection accuracy and speed, YOLOv5 emerges as the more fitting choice for this specific task.

### Model improvement experiment

In "Proposed methods" section, we introduced our proposed method, namely YOLOv5-KBS. To substantiate the efficacy of our model enhancement, we conducted ablation experiments aimed at scrutinizing the experimental outcomes. Table 4 illustrates the comprehensive experimental outcomes.

**Table 4:** Ablation experiment.

Model	K-means	SE	BiFPN	mAP50	FPS
YOLOv5l				0.757	71
	√			0.767	71
	√	√		0.771	71
	√		√	0.771	70
	√	√	√	0.799	70

As depicted in Table 4, the anchor frames generated through K-means clustering manifest as notably more fitting for the present steel surface defect detection task, leading to an enhancement in model detection result. Simultaneously, the integration of the SE attention mechanism, coupled with the substitution of the BiFPN feature fusion network, in conjunction with the utilization of the new anchor boxes, yields a discernible improvement in model performance. Ultimately, the amalgamation of all these improvement strategies, allowing them to synergistically interact, leads to a further augmentation of the model's effectiveness. The efficacy associated with the enhancement scheme put out in this article is corroborated by experimental data.

**Figure 6:**
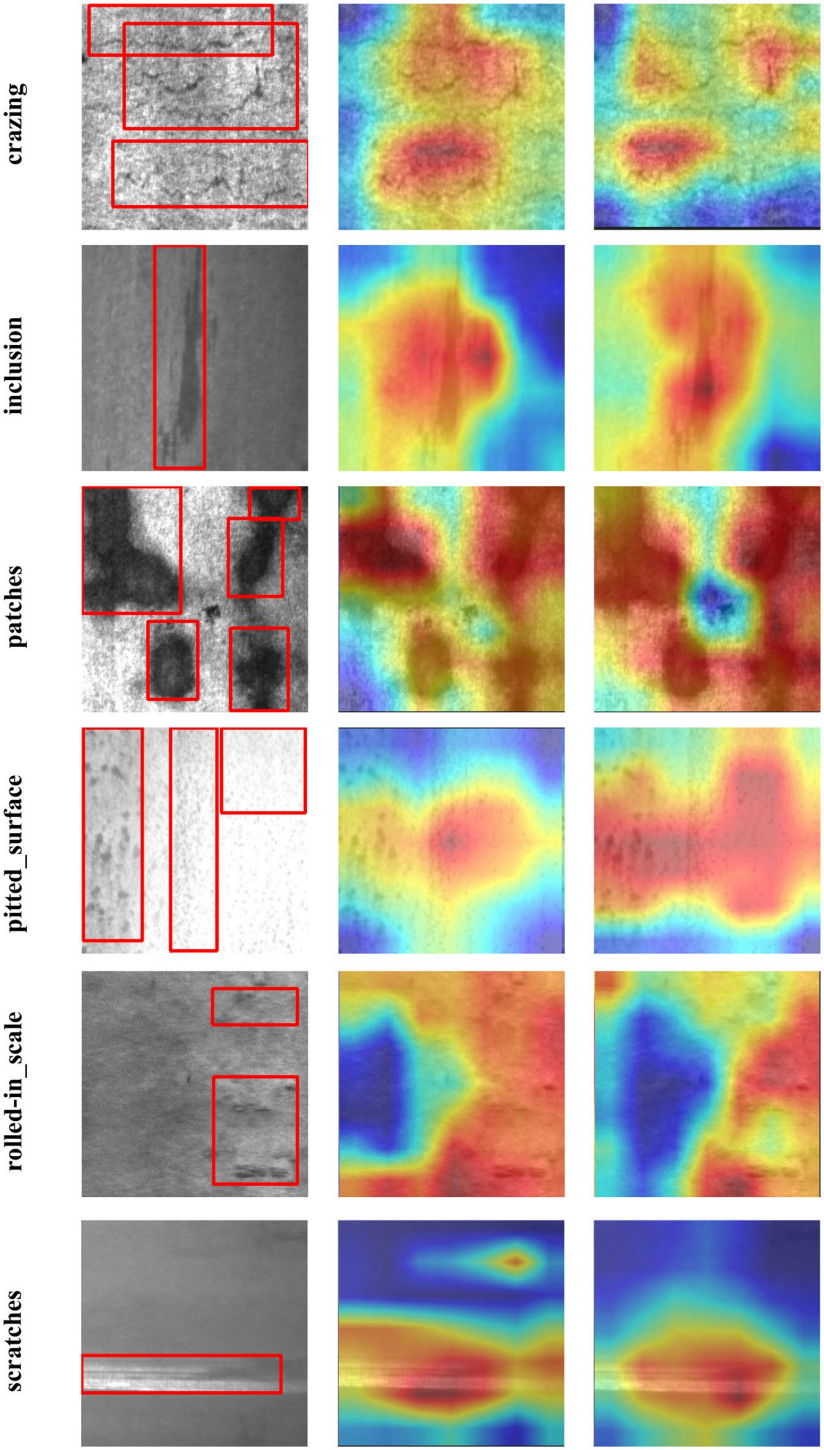
GradCAM of the model on different defects (from left to right are ground truth, YOLOv5l and YOLOv5l-SE).

To delve deeper into the influence of the SE attention mechanism, we generated the GradCAM (Gradient-weighted Class Activation Mapping)^28^ for the YOLOv5l model and the YOLOv5l-SE model which is augmented with the SE module. This enabled us to observe the specific areas of focus for each network and evaluate the impact of the SE attention mechanism on the model. The GradCAM visualizations are depicted in Fig. 6.

As depicted in Fig. 6, the incorporation of the SE attention mechanism results in an enhancement of the network's ability to focus on more pertinent information. For instance, in the case of small defects like crazing cracks, the SE module enables the model to concentrate on specific points rather than being overly fixated on the cracks and the surrounding large background area. This refinement effectively bolsters the detection proficiency for small targets. Furthermore, consider the defect of pitted_surface, characterized by patches of spots on the steel surface, which constitutes a larger target. Concentrating on a specific point within a larger object frequently results in the neglect of crucial feature. From Table 5, we can clearly see that the SE attention mechanism redirects the focus from a single point to the defective area, thereby further elevating the accuracy in detecting larger targets. In summary, the integration of the SE attention mechanism proves to be a successful enhancement.

**Table 5:** Comparison of detection accuracy of models on different defects.

Model	crazing	inclusion	patches	pitted _surface	rolled-in_scale	scratches	mAP
YOLOv5l	0.537	**0.836**	0.895	0.734	**0.668**	0.874	0.757
YOLOv5-KBS	**0.580**	0.828	**0.922**	**0.841**	0.652	**0.970**	**0.799**

Significant values are in bold.

We compared the detection accuracy of the baseline and enhanced model across various types of defects to provide a more detailed assessment of the network's detection capabilities. Table 5 presents the findings.

**Figure 7:**
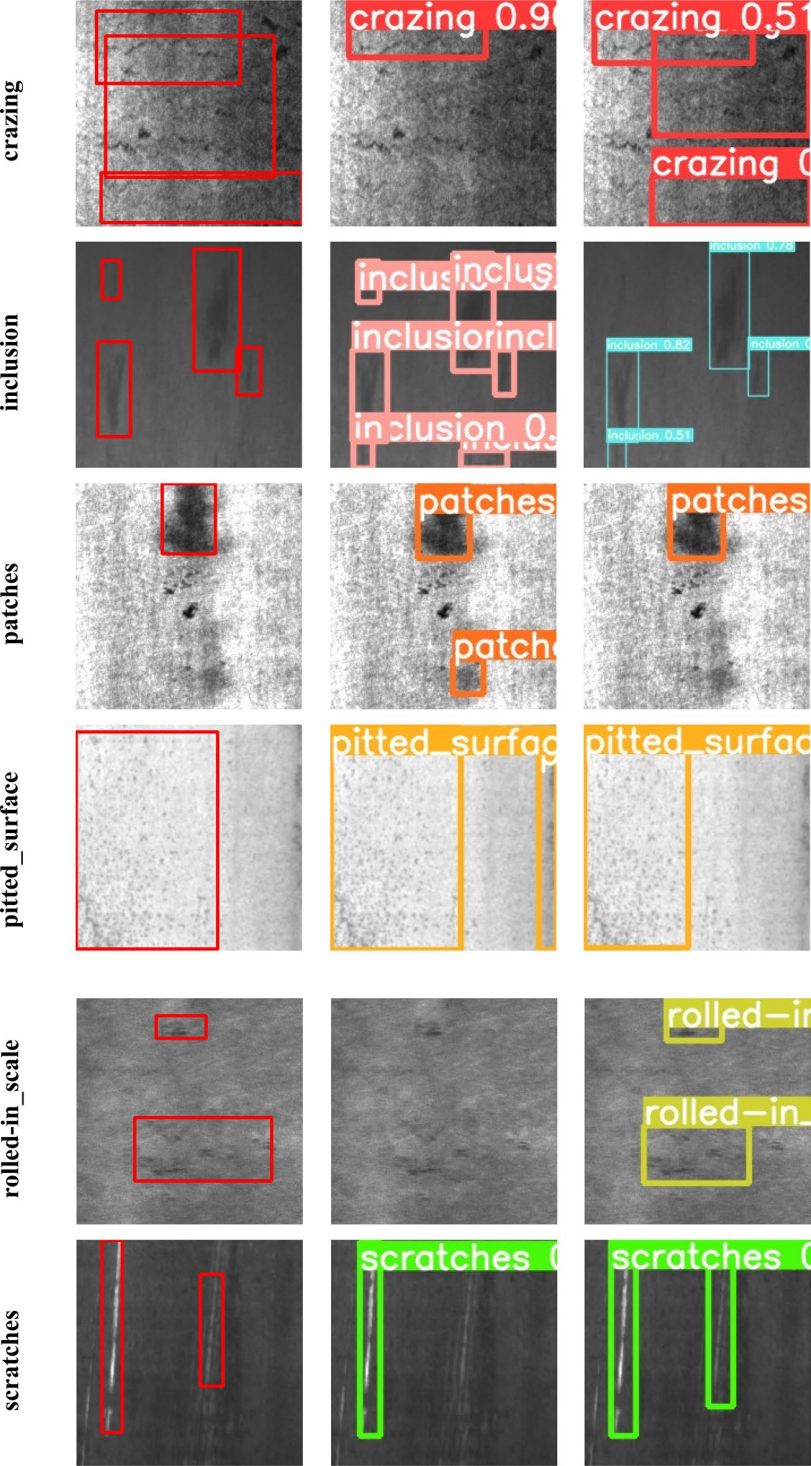
Detection results of six types of defects by different models (from left to right are ground truth, YOLOv5l and YOLOv5-KBS. Display the detected anchor box, category, and confidence level on the image).

From Table 5, The enhanced model has demonstrated substantial improvements in detecting most types of defects. Notably, the detection accuracy for scratches and pitted_surface defects has seen the most significant enhancements, with increases of 10.8% and 9.6% respectively. Additionally, the detection accuracy for crazing and patches has also witnessed notable improvements, with gains of 4.3% and 2.7% respectively. While the model does not exhibit a notable improvement in the detection of inclusion and rolled-in_scale defects, it's important to highlight that due to the model alteration, the detection accuracy for these two defect types remains nearly on par with that of the original model, indicating that the model change has not hurt their detection accuracy. The detailed detection results for different defects in the test set, as assessed by both the baseline and improved models, are outlined in Fig. 7.

From Fig. 7, it's that the original YOLOv5 model exhibited instances of both missed detection and misidentification across various defects. For instance, there were cases of missed detection in the crazing category. In contrast, YOLOv5-KBS excelled in accurately identifying all defects present in the image. In the case of patches defect detection, the original YOLOv5 model erroneously identified the background as a defect. This misclassification was effectively rectified in the improved model, demonstrating a commendable correction. On the whole, the enhanced model displays notable improvements in detecting both large and small targets without incurring a significant trade-off. Despite the increased complexity of the model, there is no substantial drop in detection speed, thereby achieving more favorable overall results in terms of performance.

## Conclusion

This research is dedicated to real-time surface defect detection during steel production. Initially, evaluate traditional inspection methods and subsequently explore deep learning approaches. Through a comparative performance analysis utilizing the NEU-DET dataset, three structural networks—YOLOv5, YOLOX, and YOLOv7—were assessed. The findings underscored YOLOv5 as the most suitable architecture for steel surface defect detection.

To further enhance detection accuracy, modifications were made to the YOLOv5l baseline model. The incorporation of SE architecture augmented focus on crucial feature channels while replacing PANet with BiFPN aimed to maximize feature fusion efficiency with reduced computational overhead. Experimental results highlight the YOLOv5-KBS model's efficacy in swift and precise real-time surface defect detection during steel production, mitigating time and labor expenses associated with defect identification. Notably, a 4.2% improvement in average accuracy over the YOLOv5l baseline was achieved. However, limitations persist, particularly in addressing overlapping flaws and detecting smaller defects such as inclusions, warranting further investigation.

## Data Availability

The datasets analysed during the current study are available at http://faculty.neu.edu.cn/songkechen/zh_CN/zdylm/263270/list/.

## References

[1] Yin X, Chen W (2013). Trends and development of steel demand in China: A bottom–up analysis. Resour. Policy.

[2] Conejo AN, Birat JP, Dutta A (2020). A review of the current environmental challenges of the steel industry and its value chain. J. Environ. Manag..

[3] Ghorai S, Mukherjee A, Gangadaran M (2012). Automatic defect detection on hot-rolled flat steel products. IEEE Trans. Instrum. Meas..

[4] Sadeghi, M. & Mahdeian, A. Application of two dimensional wavelet for defect detection in steel process. In *The 2nd International Conference on Control, Instrumentation and Automation*, 1160–1163 (IEEE, 2011).

[5] Masci, J. *et al.* Steel defect classification with max-pooling convolutional neural networks. In *The 2012 International Joint Conference on Neural Networks (IJCNN)*, 1–6 (IEEE, 2012).

[6] Soukup, D. & Huber-Mörk, R. Convolutional neural networks for steel surface defect detection from photometric stereo images. In *Advances in Visual Computing: 10th International Symposium, ISVC 2014, Las Vegas, NV, USA, December 8-10, 2014, Proceedings, Part I 10, 668–677* (Springer International Publishing, 2014).

[7] Chu M, Gong R, Wang A (2014). Strip steel surface defect classification method based on enhanced twin support vector machine. ISIJ Int..

[8] Amu, D. *et al.* Detection of wheel discoloration using R-CNN. *System*, **6** (2016).

[9] Li J, Su Z, Geng J (2018). Real-time detection of steel strip surface defects based on improved yolo detection network. IFAC-PapersOnLine.

[10] Wei R, Song Y, Zhang Y (2020). Enhanced faster region convolutional neural networks for steel surface defect detection. ISIJ Int..

[11] Tang M, Li Y, Yao W (2021). A strip steel surface defect detection method based on attention mechanism and multi-scale maxpooling. Meas. Sci. Technol..

[12] Lu-lu P, Yuan-yuan ZHU, Wen-qian JIN (2022). Surface defect detection of automotive steel parts based on improved YOLOv4J. Comput. Mod..

[13] Guo Z, Wang C, Yang G (2022). Msft-yolo: Improved yolov5 based on transformer for detecting defects of steel surface. Sensors.

[14] Chen H (2023). DCAM-Net: A rapid detection network for strip steel surface defects based on deformable convolution and attention mechanism. IEEE Trans. Instrum. Meas..

[15] Luo, Q. *et al.* CDDNet: Camouflaged defect detection network for steel surface. *IEEE Trans. Instrum. Meas.* **73**, 1–13 (2023).

[16] Li Z (2024). MPFANet: A multipath feature aggregation network for steel surface defect detection. Meas. Sci. Technol..

[17] Redmon, J. *et al.* You only look once: Unified, real-time object detection. In *Proceedings of the IEEE Conference on Computer Vision and Pattern Recognition*, 779–788 (2016).

[18] Redmon, J. & Farhadi, A. Yolov3: An incremental improvement. arXiv preprint arXiv:1804.02767 (2018).

[19] Bochkovskiy, A., Wang, C. Y. & Liao, H. Y. M. Yolov4: Optimal speed and accuracy of object detection. arXiv preprint arXiv:2004.10934 (2020).

[20] Li, C. *et al.* YOLOv6: A single-stage object detection framework for industrial applications. arXiv preprint arXiv:2209.02976 (2022).

[21] Hu, J., Shen, L. & Sun, G. Squeeze-and-excitation networks. In *Proceedings of the IEEE Conference on Computer Vision and Pattern Recognition*, 7132–7141 (2018).

[22] Tan, M., Pang, R. & Le, Q. V. Efficientdet: Scalable and efficient object detection. In *Proceedings of the IEEE/CVF Conference on Computer Vision and Pattern Recognition* 10781–10790 (2020).

[23] Lin, T. Y. *et al.* Feature pyramid networks for object detection. In *Proceedings of the IEEE Conference on Computer Vision and Pattern Recognition* 2117–2125 (2017).

[24] Liu, S. *et al.* Path aggregation network for instance segmentation. In *Proceedings of the IEEE Conference on Computer Vision and Pattern Recognition*, 8759–8768 (2018).

[25] Chen, Z. et al. Generating dynamic kernels via transformers for lane detection. In *Proceedings of the IEEE/CVF International Conference on Computer Vision* (2023).

[26] Ge, Z. *et al. *Yolox: Exceeding yolo series in 2021. arXiv preprint arXiv:2107.08430 (2021).

[27] Wang, C. Y., Bochkovskiy, A. & Liao, H. Y. M. YOLOv7: Trainable bag-of-freebies sets new state-of-the-art for real-time object detectors. arXiv preprint arXiv:2207.02696 (2022).

[28] Qiu, Z. *et al.* Is visual explanation with Grad-CAM more reliable for deeper neural networks? A case study with automatic pneumothorax diagnosis. In *International Workshop on Machine Learning in Medical Imaging*, 224–233 (Springer Nature Switzerland, 2023).

